# Effects of Children's Outdoor Physical Activity in the Urban Neighborhood Activity Space Environment

**DOI:** 10.3389/fpubh.2021.631492

**Published:** 2021-02-15

**Authors:** Yu Bao, Ming Gao, Dan Luo, Xudan Zhou

**Affiliations:** ^1^College of Horticulture, Jilin Agricultural University, Changchun, China; ^2^School of Architecture, Harbin Institute of Technology, Key Laboratory of Cold Region Urban and Rural Human Settlement Environment Science and Technology, Ministry of Industry and Information Technology, Harbin, China; ^3^School of Architecture and Urban Planning, Chongqing University, Key Laboratory of New Technology for Construction of Cities in Mountain Areas, Chongqing, China

**Keywords:** children, physical activity, environmental characteristics, spatial characteristics, neighborhood space

## Abstract

The rapid development of cities results in many public health and built-up environmental problems, which have vital impacts on children's growth environment, the development of children, and city contradictions. There is a lack of children being a main concern when constructing new urban areas or reconstructing old districts. Children's activity spaces tend to be standardized and unified (kit, fence, and carpet) “KFC style” designs, which leads to the urban neighborhood space and the environment being insufficient to attract children to conduct activities. Therefore, starting from the urban neighborhood space environment, this paper explores what kind of spatial environment is needed for children's physical activity and its impact on children's physical activity. Taking six residential areas in the Changchun Economic Development Zone as the research object, based on the theory of children's ability development and game value, this paper uses the Woolley and Lowe evaluation tool to quantify the impact of the theory on the urban neighborhood space environment and children's physical activity. It can be confirmed that there is a significant correlation between the spatial characteristics of an urban neighborhood and the general signs of the environment on the duration and intensity of the physical activity of children. The results show that: (1) the differences in children's ages result in differences in the duration and intensity of children's physical activity in the urban neighborhood space environment; (2) the open space factor of the neighborhood space has the most significant influence on the duration of children's physical activity; (3) in terms of the environmental characteristics, whether children can be provided with education and learning opportunities has a significant impact on the duration of children's physical activity; (4) there is a significant positive correlation between children's age and the duration and intensity of the physical activity, exercise type, and imaginative activity. These results show that the urban neighborhood space environment can affect the duration of children's physical activity. In future urban residential area planning and design, urban children can meet the self-demand of physical activity in the neighborhood space through the reasonable balance and combination of neighborhood space characteristics and environmental characteristics.

## Introduction

A large number of studies have focused on the link between child health problems and environmental characteristics, such as the impact of neighborhood spatial security on physical activity (1), and the report on the impact of low-income neighborhoods on youth physical activity (2). It mainly follows the logic of “spatial demand-influencing factor-planning strategy” (3, 4), and makes a systematic study on the demand and planning strategy of urban children's outdoor public space (5, 6). Western countries that have experienced earlier urbanization are gradually aware of the negative impact of rapid urbanization on children's physical and mental development. High-rise and high-density urban development has squeezed children's public activity spaces, such as outdoor playgrounds and green spaces (7, 8). The independent activity and physical activity levels of children in outdoor public activity spaces have decreased sharply in many countries (9–11). However, childhood obesity, cardiovascular disease, and psychological disease have increased significantly each year (12). A lack of physical activity is one of the leading causes of children's health problems. Health environment research has a long history (13, 14). In recent years, the neighborhood environment's impact on health, which is a microspatial scale effect, has received little attention (15, 16). Neighborhood space, as the closest to life space category in residents' daily lives, is also the primary place for children's daily activities, and thus the neighborhood space has a broad impact on children's physical and mental health (17, 18). According to the theory of children's ability development, Woolley defined the developmental themes of children's activity space as “environmental,” “physical logical,” “social,” “creative,” and “educational.” In outdoor activity spaces, children's social, emotional, cognitive, and mental health development can be promoted through game activities (19–22).

The opportunity to promote physical activity in a neighborhood environment means creating the environment. Through environmental construction, mobility can be reduced to promote deep-seated interaction and communication. The environmental perception is people's subjective feelings and psychological judgements of the surrounding environment and its changes and is the psychological basis of people's environmental behavior. Only through the cognitive environment can human beings guide their behavior through the environment. Children generate information via landscape stimulation through perception, generate cognition, and form a corresponding emotional reflection, which will affect the degree of engagement of children in physical activities. It is essential to understand the relationship between the neighborhood environment, children's physical activity, and their growth and development. The rationality and scientificity of neighborhood environmental activity space planning and design from children groups' perspectives can effectively promote children's physical activity. They will affect children's behavioral mode and health status in the future (23, 24), which are very important for their healthy growth.

With the rapid development of urbanization, urban construction has improved rapidly. Nevertheless, the development of children's playgrounds is relatively backward, and they are not adequately planned and allocated, reflecting the contradiction between children's needs and the urban development. The allocation of public resources in a fair and just society must be based on maximizing the interests of the weakest. As the lowest level of many groups, children should be the group that the government pays the most attention to when formulating public policies (25). Children's playground design tends to be instrumental and indoor as a socially vulnerable group due to their fundamental demands that are not easy to express (26, 27). For children, the urban outdoor activity spaces are hugely compressed. As children interact most closely, the physical activity status of urban neighborhood spaces has become an urgent research topic (28). To address the particularities of children groups, the security problems and responsibility of children's playgrounds are also reasons that children's playgrounds excessively rely on finished game facilities. Moreover, the spatial environment around children's residences is incredibly closely related to children's physical activity (29, 30), and the global positioning system shows that 63% of children's physical activity is near their residences (31). It has been shown that the community neighborhood space environment is the most essential outdoor activity space for children, and the space's environmental quality has also become the most critical factor affecting children's physical activity (32). Besides, Western countries mainly focus on single-factor investigations from the perspective of hygiene, focusing on children's health and physical activity levels.

This study was conducted to investigate the physical activities of children in the neighborhood space. Starting with the social attributes of children, this paper analyses the correlation between children's physical activity types and the spatial factors in their neighborhood and environmental factors to explore the needs of children's activities for the spatial environment.

Therefore, based on the theory of children's psychological cognitive development, the relationship between the urban neighborhood space environment and children's physical activity is quantified. Specifically, our aim is to explore the influence of the neighborhood space and children's physical activity from a microdesign perspective by quantifying the physical activities of children in neighborhood spaces.

In this work, we ask the following research questions:

Research question 1 (RQ1): Does the difference in children's ages affect the duration and intensity of physical activity in urban neighborhood spaces?
Research question 2 (RQ2): Among the spatial characteristics and environmental characteristics of urban neighborhoods, such as vegetation, are there relevant factors affecting the duration and intensity of children's physical activity? What factors are related to the duration and intensity of children's physical activity, and which factor has the most significant impact on the factors affecting the duration and intensity of physical activity?
Research question 3 (RQ3): Does the type of physical activity in children affect the conclusions already observed?

## Methods

### Scope of Study

**Figure 1:**
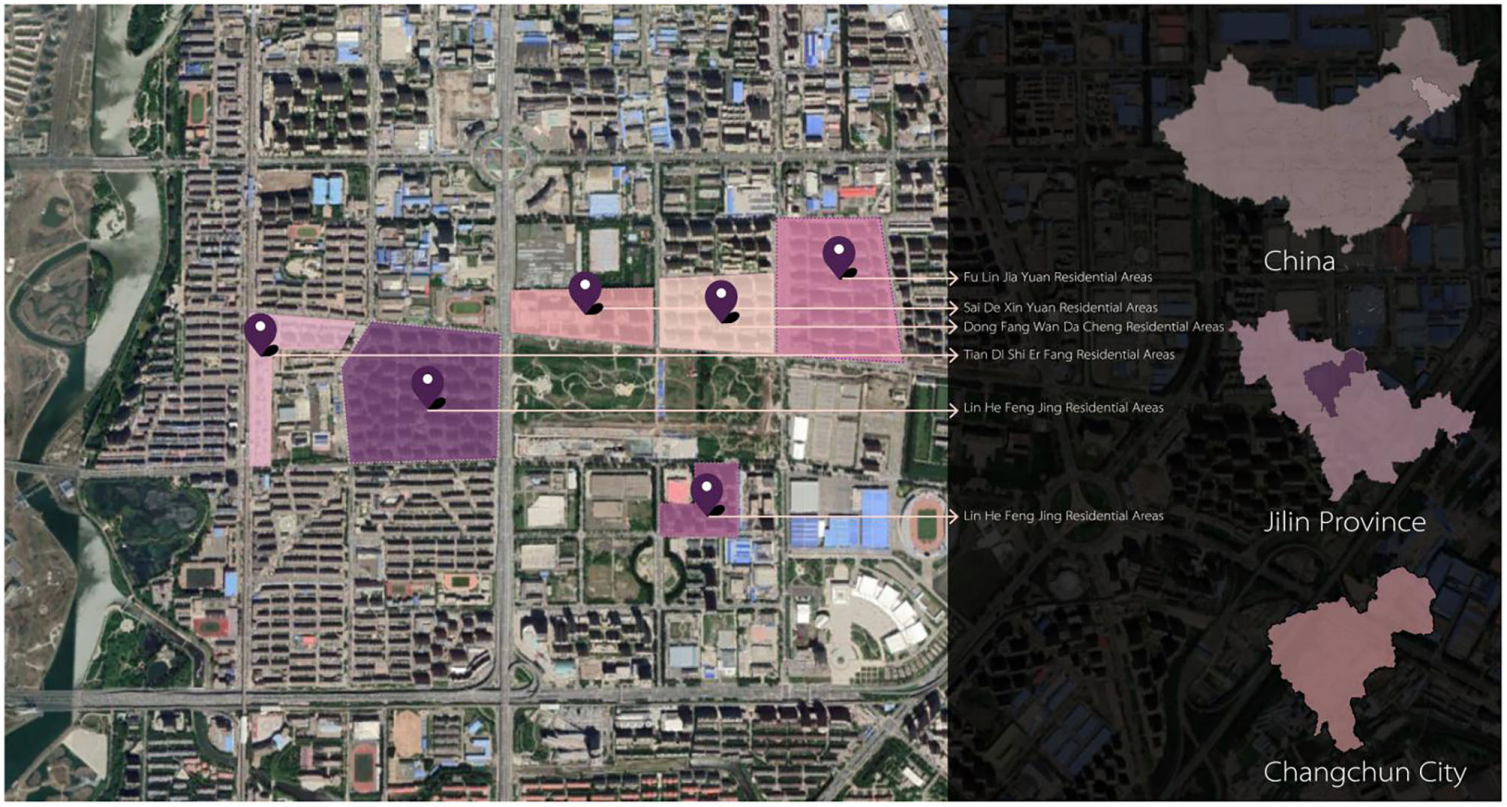
Location and scope of the study.

In domestic research, a “neighborhood” has no clear regional boundary in terms of spatial elements, so it usually refers to a neighborhood space defined by the community's administrative division. In the investigation and visiting process, it was found that the residents of each community do not flow frequently, and most of the activities are concentrated in the residential area. Therefore, compared with the community, the residential area is more in line with the concept of a “neighborhood” (15). In this study, the residential area is taken as the spatial unit of neighborhood research, and six residential areas of the Changchun and Technological Development Zone are selected as the research objects, as shown in Figure 1. They are the following: Fulin Jiayuan, Sade Xinyuan, Dongfang Wandacheng, Linhe Fengjing, Tiandi Shierfang, and Beihai Lijing. There are many residents and children here, and the residential area was built at an exact time. It has social and economic characteristics, reflecting the physical space environment surrounded by urbanization (28). Therefore, the research will help to obtain universal research results.

### Research Method

Through field visits, the spatial notation method (33, 34) was used to observe the situation and form of children's physical activity in the neighborhood environment activity spaces, and the assignment method was adopted to assess the intensity of physical activity. First, children's activity intensity was divided into five levels: low, lower, medium, higher, and high. The values ranged from 1 to 5 (35) from low to high, respectively. The average value of children's physical activity intensity indicated that children were living. All types of activities occur in busy times. This value is relative. The higher the value is, the higher the intensity of the physical activity. Second, a questionnaire was designed to investigate the types and characteristics of children's physical activity in the study area, and the Woolley and Lowe assessment tool was used to evaluate the spatial characteristics and environmental characteristics of the neighborhood outdoor space (37). Based on the concept of children's activity game value and the research from many fields, the British researcher Woolley (38) proposed corresponding evaluation tools based on the relationship between children's space design and the activity value.

In the preliminary survey, it was found that most of the children who play independently on outdoor playgrounds do so most often on weekends. Therefore, this survey focuses on the weekend. During this period, there were large numbers of children and sufficient samples. After asking parents' permission and the child's consent we started these research activities. We explained our organization and identity to the interviewees (certificates and letters of introduction issued by the school), and ensured that all the information of the interviewees would only be used for this academic research and would never be disclosed through any means which made the interviewees fully trust our research team and then they authorized the research. Besides, fill in the questionnaire. We explained the reason and purpose of our research which aimed at exploring the influence of environmental and space on children's activities. Meanwhile, only one respondent was surveyed to elaborate and explain the problem. Therefore, both the children themselves and their guardians are willing to provide information, and the respondents' confirmation of the authorization of information is included in each questionnaire. During the questionnaire distribution, we distributed questionnaires one by one, filled them out, collected them, and then issued the next questionnaire which needed a number of volunteers conducting research together. In this way, the recovery rate of the questionnaire is guaranteed. At the same time, if the recruiters do not understand the contents of the questionnaire when filling in the questionnaire, the volunteers can give immediate and timely reply, which ensures the efficiency of the questionnaire. A total of 510 questionnaires were distributed, and 500 were recovered. Of these 500 questionnaires, which 498 were valid, and the effective response rate was 97%. The questionnaire content involves children's age, time, frequency, activity type, and evaluation of the spatial and environmental characteristics of children's activities in residential areas.

### Research Object

Due to children's limited cognitive ability, only 4- to 15-year-old children were investigated and divided into three age groups: 4–7, 8–11, and 12–15 years old. The selection of the children as the research objects is based on the following two reasons: (1) According to the cognitive development theory of child psychologist Jean Piaget (36), the developmental concept of the interaction between children's psychological development and environment (37) defines this as appropriate, and children's physical and mental characteristics are shown in Table 1. (2) Four- to 15-year-old children have relatively healthy independence, and they have more opportunities and types of physical activities in which to participate. Game activities are the main activities of children in the age group, and they have a clear image and initiative in the process of playing (26). The diversity of activity types is more abundant, they can express their wishes, and the influence of guardians on children's activities is small (39). Children aged 8–11 and 12–15 were interviewed face-to-face, and questionnaires were used. Parents or child caregivers completed the questionnaires reviewing the neighborhood space's physical activities for the children aged 4–7.

**Table 1:** Characteristics of children's psychological development and their environmental needs.

**Children's cognitive development stage**	**Characteristics of children's psychological development**	**Characteristics of children's activity environment**
2–6 years old: Preoperational stage	Internalization of perceptual action into representation	Color, patterns, and materials are needed to stimulate children's perception of things
7–11 years old: Concrete operations stage	Think logically about things	The connection and division of space is needed to meet children's social and private contact needs
12–15 years old: Formal operational stage	The ability to conduct abstract thinking	Approaching adult needs

### Reliability and Validity

Statistical Package for the Social Sciences (SPSS 20.0) was used to process the data to analyse the validity and reliability of urban neighborhood spatial environmental activities. The factors involved in the questionnaire were standardized with SPSS 20.0, and then reliability analysis was conducted. Reliability analysis was used to study the reliability and accuracy of quantitative data. When analyzing the α coefficient of the questionnaire (Cronbach's α coefficient), if this value is higher than 0.8, it indicates high reliability. The Cronbach's α coefficient of this study is 0.841. The KMO and Bartlett test are used to verify the validity. The KMO is 0.801 and >0.8. Thus, the validity of the research data is excellent. Therefore, it can be inferred that the questionnaire's validity and reliability are promising, indicating that the data are real and practical.

## Results

### Data Statistics

**Figure 2:**
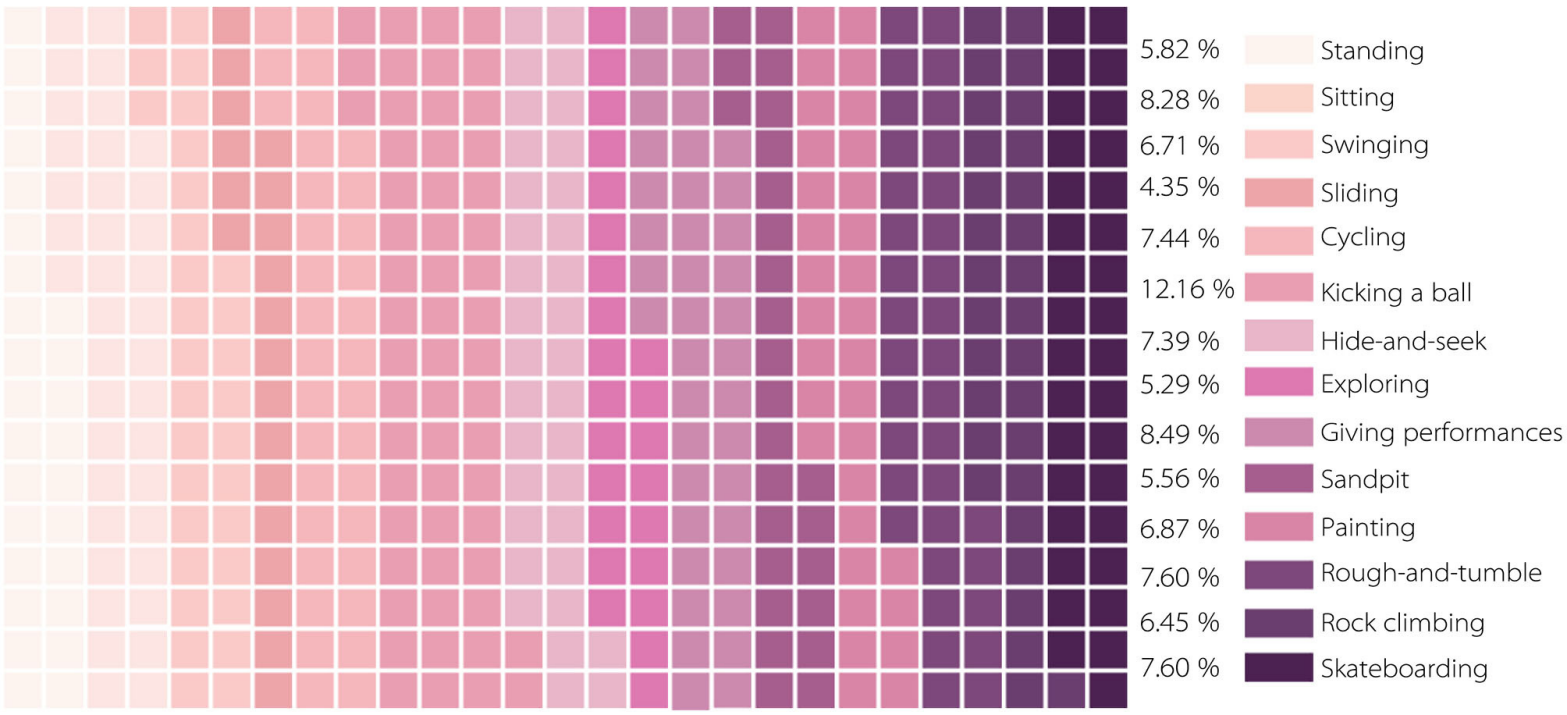
Proportion of physical activity types in children.

As shown in Table 2, regarding age, the age frequency distributions of the three sample groups are not significantly different, which shows that the age distribution of children in urban neighborhood space activities is relatively uniform. Nearly one-third of the children in the sample had an activity duration of “more than 90 min,” accounting for 29.80% of the total. In terms of physical activity intensity, 61.20% of children's activity intensity was at high and medium-high levels. Furthermore, 39.00% of the activity time was in the “afternoon.” Besides, the proportion of evening samples was 38.20%, and more than half of the activity time was in the afternoon and evening. Additionally, the types of children's physical activities were statistically analyzed, as shown in Figure 2. We found that in the neighborhood space, the activity frequency of kicking a ball was the highest. The frequency of slide activity was the lowest; and the frequencies of rock climbing, swinging, playing hide and seek, painting, skateboarding, cycling, and the five other types of activities were not significantly different.

**Table 2:** Analysis of children's age, activity period, activity duration, and intensity.

**Name**	**Option**	**Distribution**	**Percentage (%)**	**Cumulative percentage %**
Age (years)	12–15 years old	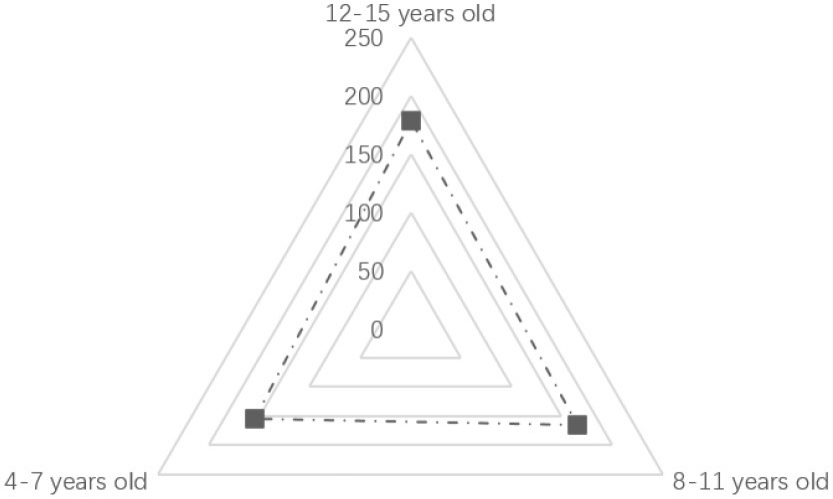	35.80	35.80
	8–11 years old		33.20	69.00
	4–7 years old		31.00	100.00
Activity period	Forenoon	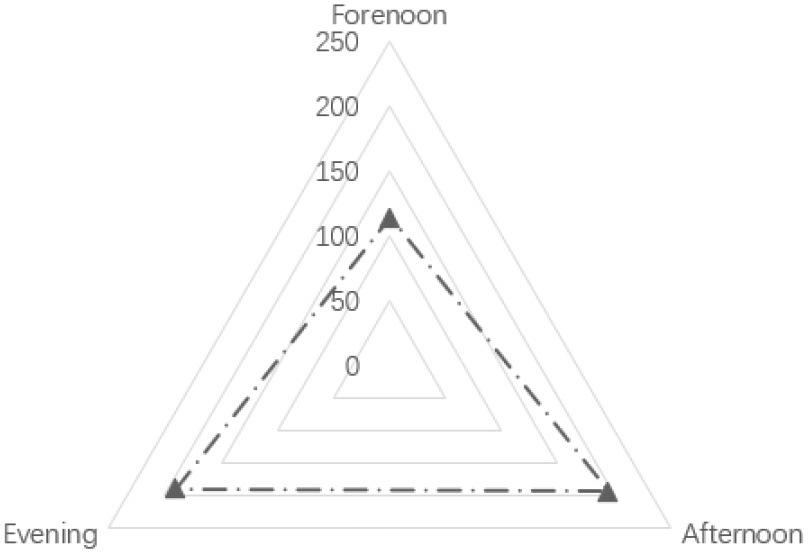	22.80	22.80
	Afternoon		39.00	61.80
	Evening		38.20	100.00
Activity duration (min)	21–40	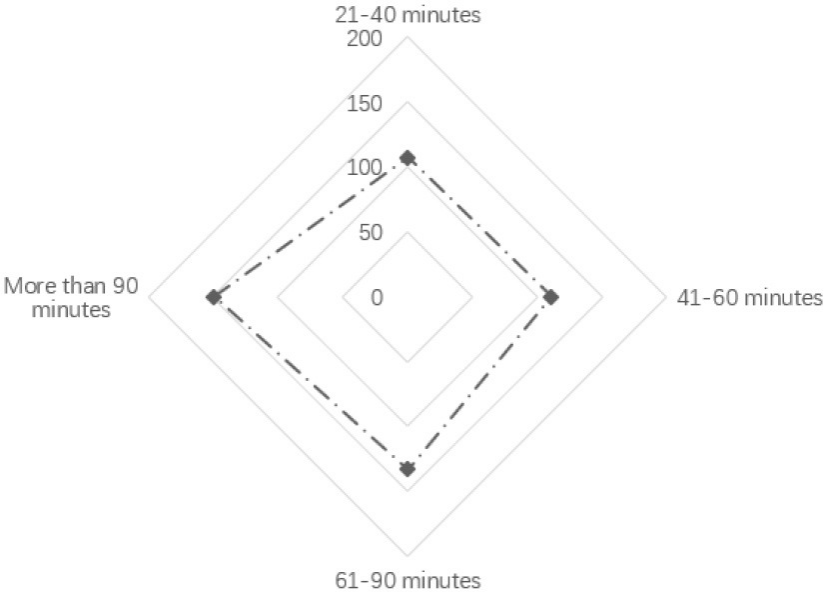	21.40	21.40
	41–60		22.20	43.60
	61–90		26.60	70.20
	>90		29.80	100.00
Physical activity intensity	Low (0 < intensity < =1)	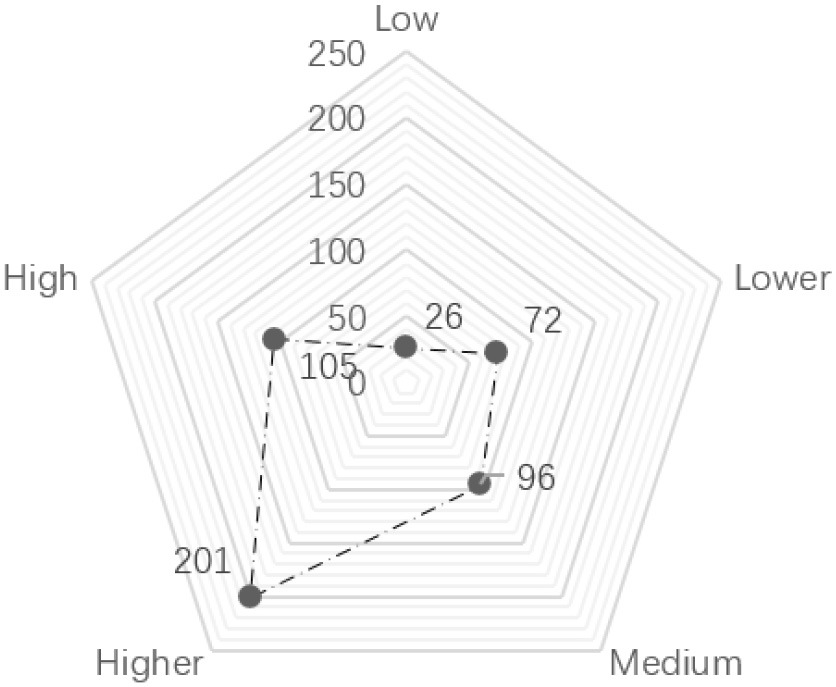	14.40	19.60
	Lower (1 < intensity < =2)		19.20	38.80
	Medium (2 < intensity < =3)		40.20	79.00
	Higher (3 < intensity < =4)		21.00	100.00
	High (4 < intensity < =5)		14.40	19.60

### Children's Physical Activity and Neighborhood Spatial Relationship

In this section, we review the research issues. Analysis of variance (single-factor analysis of variance) was used to study the difference between children's age and the duration and intensity of physical activity. Therefore, we can answer research question 1 (RQ1). Table 3 shows that different age samples have a significant effect on the duration and intensity of physical activity (*p* < 0.05), which means that different age samples have significant effects on the different times and intensities of physical activity.

**Table 3:** Analysis of variance between the duration of children's physical activity and children's age.

	**ANOVA**				
	**Age (years) (mean ± SD)**			**F**	***p***
	**12–15 years old (*n* = 179)**	**8–11 years old (*n* = 164)**	**4–7 years old (*n* = 155)**		
Activity duration (min)	3.26 ± 0.90	2.68 ± 1.06	1.92 ± 0.98	78.981	0.000^**^
Physical activity intensity	4.17 ± 0.65	4.20 ± 0.61	2.23 ± 0.75	455.284	0.000^**^

* When the confidence value (double test) is 0.05, the correlation is significant;

** *When the confidence value (double test) is 0.01, the correlation is significant*.

To answer research question 2, we calculated the correlations of the time and intensity of physical activity and the relationship between the intensity and the surrounding spatial environment. The Pearson correlation coefficient was used to identify the strengths and weaknesses of the correlation. Table 4 shows the results for the length of children's activities and the open space, different scales and types of space, vegetation, and the spatial characteristics of children's activities. There is a positive correlation between the physical boundary, and playable water and sand at a site. The physical activity intensity of children shows a negative correlation with the terrain, movable materials, the physical boundary of the site, and playable water and sand, which shows that these factors limit children's medium- and high-strength physical activities.

**Table 4:** Correlation analysis of the duration and intensity of children's physical activity and neighborhood spatial factors.

**Pearson correlation**			
		**Activity duration (min)**	**Physical activity intensity**
Range of fixed play equipment	Correlation coefficient	−0.019	0.388^**^
	*p*	0.674	0.000
Open spaces	Correlation coefficient	0.116^**^	0.131^**^
	*p*	0.010	0.003
Different sizes and types of spaces	Correlation coefficient	0.109^*^	−0.074
	*p*	0.014	0.100
Vegetation	Correlation coefficient	0.151^**^	0.168^**^
	*p*	0.001	0.000
Landform	Correlation coefficient	−0.009	−0.099^*^
	*p*	0.840	0.027
Moveable equipment	Correlation coefficient	−0.026	−0.092^*^
	*p*	0.560	0.041
Obvious physical boundaries	Correlation coefficient	0.107^*^	−0.094^*^
	*p*	0.017	0.037
Seating opportunities	Correlation coefficient	−0.069	−0.036
	*p*	0.122	0.417
Surface materials	Correlation coefficient	0.067	0.007
	*p*	0.132	0.883
Water and sand	Correlation coefficient	0.097^*^	−0.258^**^
	*p*	0.031	0.000
Natural materials	Correlation coefficient	−0.010	−0.047
	*p*	0.829	0.291

* *p < 0.05*,

** *p < 0.01*.

### Relationship Between Children's Physical Activity and Neighborhood Environment

The Pearson correlation coefficient was used to identify the strength of the correlation relationship between children's physical activity time and the neighborhood environment, as shown in Table 5. Among the factors related to children's physical activity time and the neighborhood environment, physical activity time was significantly negatively correlated with whether visual stimulation was used and whether education and learning opportunities were provided. This is a different answer to research question 2 (RQ2). Regarding the intensity of physical activity, whether the environment is challenging has a positive correlation and whether the space is attractive has a negative correlation with the intensity of activity.

**Table 5:** Correlation analysis of the duration and intensity of children's physical activity and neighborhood environmental factors.

**Pearson correlation**			
		**Activity duration (min)**	**Physical activity intensity**
Is the area enticing?	Correlation coefficient	0.061	−0.197^**^
	*P*	0.174	0.000
Is the area stimulating?	Correlation coefficient	−0.091^*^	0.011
	*P*	0.041	0.809
Is the area challenging?	Correlation coefficient	−0.048	0.223^**^
	*P*	0.285	0.000
Are there learning opportunities?	Correlation coefficient	0.155^**^	−0.065
	*P*	0.000	0.148
Is the area appropriate for all age groups?	Correlation coefficient	−0.182^**^	0.007
	*P*	0.000	0.868

* *p < 0.05*,

** *p < 0.01*.

### The Relationship Between Children's Physical Activity, Age, and Neighborhood Spatial Activity Types

Research question 3 (RQ3) questioned whether the type of physical activity would affect the duration and intensity of physical activity. As shown in Table 6, we analyzed the correlation between 14 types of children's physical activity. (1) The length of children's activities has a significant positive correlation with the types of activities, such as adventuring, performing programmes, and painting; and a negative correlation with the types of activities such as standing, sitting, and playing in a sandpit. (2) As for the intensity of the physical activity, it was negatively correlated with activities such as standing, sitting and playing in a sandpit and was positively correlated with cycling, playing football, playing hide and seek, exploring, giving performances, painting, rough-and-tumble, rock climbing, skateboarding, etc. Besides, we add that there is a significant positive correlation between children's age and swinging, sliding, cycling, playing hide-and-seek, exploring, playing in a sandpit, skateboarding, and other activities and negatively correlated with giving performances, painting, rock climbing, rough-and-tumble, and other activities.

**Table 6:** Correlation analysis of the types of children's activities.

**Pearson correlation**				
		**Activity duration (min)**	**Physical activity intensity**	**Age**
Standing	Correlation coefficient	−0.262^**^	−0.713^**^	0.043
	*p*	0.000	0.000	0.341
Sitting	Correlation coefficient	−0.216^**^	−0.576^**^	0.000
	*p*	0.000	0.000	0.992
Swinging	Correlation coefficient	0.053	0.035	0.171^**^
	*p*	0.233	0.438	0.000
Sliding	Correlation coefficient	−0.013	0.002	0.166^**^
	*p*	0.765	0.970	0.000
Cycling	Correlation coefficient	−0.067	0.440^**^	0.351^**^
	*p*	0.132	0.000	0.000
Playing football	Correlation coefficient	0.053	0.566^**^	0.078
	*p*	0.241	0.000	0.084
Playing hide-and-seek	Correlation coefficient	0.065	0.169^**^	0.385^**^
	*p*	0.144	0.000	0.000
Exploring	Correlation coefficient	0.158^**^	0.297^**^	0.380^**^
	*p*	0.000	0.000	0.000
Giving performances	Correlation coefficient	0.257^**^	0.301^**^	−0.538^**^
	*p*	0.000	0.000	0.000
Playing in a sandpit	Correlation coefficient	−0.213^**^	−0.295^**^	0.173^**^
	*p*	0.000	0.000	0.000
Painting	Correlation coefficient	0.179^**^	0.096^*^	−0.331^**^
	*p*	0.000	0.031	0.000
Rough-and-tumble	Correlation coefficient	0.320^**^	0.204^**^	−0.543^**^
	*p*	0.000	0.000	0.000
Rock climbing	Correlation coefficient	0.093^*^	0.477^**^	−0.310^**^
	*p*	0.039	0.000	0.000
Skateboarding	Correlation coefficient	0.064	0.395^**^	0.229^**^
	*p*	0.155	0.000	0.000

* *p < 0.05*,

** *p < 0.01*.

The types of children's physical activity are relatively diverse. Referring to the theory of availability and the description of children's physical game classification by Fjørtoft (3), the 14 types of children's physical activity are divided into four types: passive, physical, constructive and symbolic. They are shown in Table 7.

**Table 7:** Classification of children's activity types by Fjørtoft.

**Activity type**	**Characteristics**	**Give an example**
Passive type	Related to more than children's visual perception, and the intensity of the physical activity is very low.	Sitting and standing
Sport type	Related to more than children's physical exercise; more intense physical activity; and less skill and creativity.	Running and rolling, cycling, rock climbing, sliding, swinging, playing football, playing hide and seek, exploring, etc.
Constructive	Children use, combine, and divide some mobile environmental factors to conduct the activity, and the intensity of the physical activity is moderate.	Playing with sticks and pebbles, building small Peng houses, piling sand, etc.
Imaginative	Children use their imagination to conduct some imitative and expressive activities. The intensity of physical activity varies from moderate to severe.	Role playing, performing, etc.

As shown in Table 8, correlation analysis was used to study age; activity duration and intensity; and the passive, athletic, and imaginative types. The Pearson correlation coefficient was used to measure the strength of the correlation. The results showed that (1) there was a significant positive correlation between children's age and sports activities and a significant negative correlation between children's age and imaginative and constructive activities. (2) There was a significant positive correlation between physical activity and motor and imaginative activities and a significant negative correlation between the durations of physical activity and passive activity. (3) There is a positive correlation between the intensity of physical activity and the type of exercise and imagination. There is a significant negative correlation between the intensity of physical activity and the passive type and the construction type.

**Table 8:** Correlation analysis of children's activity types.

**Pearson correlation**				
		**Activity duration (min)**	**Physical activity intensity**	**Age**
Passive type	correlation coefficient	−0.262^**^	−0.713^**^	0.043
	*p*	0.000	0.000	0.341
Sport type	correlation coefficient	0.196^**^	0.675^**^	0.233^**^
	*p*	0.000	0.000	0.000
Constructive	correlation coefficient	0.252^**^	0.300^**^	−0.544^**^
	*p*	0.000	0.000	0.000
Imaginative	correlation coefficient	−0.016	−0.151^**^	−0.144^**^
	*p*	0.725	0.001	0.000

* When the confidence value (double test) is 0.05, the correlation is significant;

** *When the confidence value (double test) is 0.01, the correlation is significant*.

## Discussion

### Results of the Evaluation Tools

In previous research, we also studied the correlation between urban outdoor space and children's emotional and physical activities (11). The fairness of urban built-up environments for children is not high. The care given to children is insufficient (40). This study's main purpose is to explore the relationship between the spatial environment of the urban neighborhood and children's physical activity. The research shows that the relationship between the neighborhood's spatial characteristics and the physical activity of children can be evaluated following Woolley and Lowe. However, the evaluation results of the Woolley and Lowe assessment tool can objectively reflect the relationship between children's activities and space and show that there is a significant correlation between them. The results show a positive correlation between sports and constructive activities and children's age while there is a negative correlation between passive activities and the duration of children's activity. In terms of the duration and type of physical activity of children, the diversity of the spatial environment and spatial factors in urban residential areas promotes children's physical activity level. Simultaneously, it also provides more opportunities for children to choose their own preferred and suitable physical activities, enhances children's interest in activities, and reduces the possibility of children participating in passive physical activities (41). Besides, the influences of vegetation and the development space's elements on children's physical activity time are the most and second most important characteristics of neighborhood space; therefore, it is particularly important to ensure the equalization of children's activity space and green space vegetation in neighborhood space.

### The Contradiction of Children's Activity Space

Since children's activity space tends to be a kit, fenced in, or PET, the excessive artificial design and uniform equipment and facilities in the activity field lack consideration of children's needs, limiting the level of children's outdoor physical activity. Therefore, the goal of the equalization of neighborhood space is to meet children's activities, emotional communication, physical development, etc. From the microdesign point of view, different from the previous study on whether the urban residential neighborhood space is child-oriented that showed that the main criteria include accessibility, safety, and employability (42), this study explored and analyzed the relationship among children's physical activity, spatial environmental factors, and children's physical activity types based on the spatial characteristics, environmental characteristics, and types of children's physical activity in an urban neighborhood. The diversity of neighborhood space environmental factors has a positive impact on the duration of children's physical activity, improving the quality of children's physical activity and allowing children to acquire the five abilities of “physical,” “social,” “emotional,” “creative” and “challenging” at the same time (43). The diversity also enables children to obtain pleasant feelings and rich experiences through physical activities, thus improving children's activities in the neighborhood space environment (43). In previous studies, we also confirmed that natural materials such as vegetation have a significant correlation with the duration of children's activity in the spatial environment. They can promote the duration of and intensity children's physical activity, and children with longer outdoor activity times have higher cardiopulmonary fitness (44) and better children's physical and mental health development (45, 46). However, there are few children's activity places with natural materials in the urban neighborhood space, which shows a significant deviation between children's own needs, the actual construction of the city and the habitual neglect and deprivation of children's rights (25).

### Enlightenment on Children's Activity Strategy

Neighborhood space and its environmental elements play potential roles in children's activities, growth, and development. Based on the theory of mind (TOM) (47, 48), we can exchange our ideas, goals, and desires with others in activities and promote children's ability to make friends and socialize. Creating ambiguity in the spatial environment will enable children to explore more (49), which is the site's physical boundary index mentioned in the Woolley and Lowe evaluation tool. Therefore, the construction and design of children's outdoor space activity places need to consider children's unique attributes. Based on the interdisciplinary theories of children's psychology, environmental behavior, cognitive science, and development psychology (36), the national government should promote children-based policies and improve the construction of public space for children.

### Limitations of Research

Based on the main relationship between the spatial environment and children's activity level, the results for the urban neighborhood spaces of other cities may be different due to economic and regional differences. Besides, most of the current relevant research on children's outdoor space design results from the perspective of children's psychological and physiological needs explores children's space needs at different ages and children's behavior development in different spaces while the research on activity intensity is relatively limited. It is hoped that our research can be used deeply explore the level of physical activity of children of different ages in different cities. This study is based on field surveys, questionnaire interviews, and direct observations. Although these qualitative survey data can be quantified, there is still a lack of instruments to detect the objective neighborhood space physical environment index and its impact on children's physical activity opportunities (9). Future research should be quantified using the objective indicators and the research precision and depth should be deepened.

Besides, we use Woolley & Lowe assessment tools in the environment and the space of two dimensions scores to add together in order to achieve the final evaluation score. Not only this way of statistics in statistical is easier to obtain the relationship between the two kinds of things but also the evaluation criterion that are included in the tool are applicable.It also can make more specific description for the environment and space specific, cause the angel to clearly understand of the meaning of the questionnaire, and let the results will be more credible. For the impact between the environment space and children's activities discussed in our study, it is more appropriate, and it is advantageous for the adynamic place of children and the score of value. This is of great reference significance to the establishment of evaluation criteria for the design of children's activity space. However, this evaluation tool originated in the UK, and to some extent has been influenced by the UK. If this tool is to be more widely used in the future, some evaluation criteria need to be adjusted and optimized.

## Conclusions

The urban neighborhood space environment has an important impact on urban residents' physical and mental health, including children. This kind of neighborhood space environment in people's daily lives will affect basic well-being, such as health and safety, and the learning ability and development level of children.

In this study, the neighborhood space environment was used to explore the duration of children's physical activity, to study public space planning and design needs, and to ensure the allocation of children's activity space in the public space. Different from the traditional choice of the residential area surrounding environmental variables as the research area, we use the perspective of children to explore the impacts of the space and environment on children's physical activity and to explore the influence of several different characteristics on children's physical activity level. The aim is to shape and improve outdoor public space, create high-quality outdoor opportunities for children, make children healthier and more creative, and better integrate children into society.

First, regarding neighborhood space characteristics, vegetation landscape elements can provide visual aesthetic and interactive opportunities for children in outdoor activity spaces. There is a significant positive correlation between the duration of children's activities and the intensity of physical activities. Open space provides places for children's activities, group activities, and group activities, increasing the opportunities for children's extracurricular activities, thereby promoting children's physical activity level. Second, children's physical activity time can be prolonged by contacting the natural environment in the neighborhood space and providing children with the opportunity for operations or experimentation. Finally, regarding the types of children's activities, passive activities shorten the time of children's physical activities and limit the intensity of children's physical activities.

## Data Availability Statement

The datasets presented in this article are not readily available because we explained our institution and identity (certificate and letter of introduction issued by the University) to the interviewee. We ensured all the interviewee's information was only used for academic research and would not be disclosed in any way, which made the interviewee fully trust the research team and then authorized the research. Requests to access the datasets should be directed to 19S034123@stu.hit.edu.cn.

## Ethics Statement

Ethical review and approval was not required for the study on human participants in accordance with the local legislation and institutional requirements. Written informed consent to participate in this study was provided by the participants' legal guardian/next of kin.

## Author Contributions

Conceptualization: YB and MG. Methodology, writing—original draft preparation, and visualization: MG. Software, investigation, and data curation: YB. Validation and formal analysis: MG and DL. Resources and supervision: YB, XZ, and DL. Writing—review and editing: MG, XZ, and DL. Funding acquisition: DL. All authors have read and agreed to the published version of the manuscript.

## Conflict of Interest

The authors declare that the research was conducted in the absence of any commercial or financial relationships that could be construed as a potential conflict of interest.
